# Local and Systemic Alterations of the L-Arginine/Nitric Oxide Pathway in Sputum, Blood, and Urine of Pediatric Cystic Fibrosis Patients and Effects of Antibiotic Treatment

**DOI:** 10.3390/jcm9123802

**Published:** 2020-11-24

**Authors:** Beatrice Hanusch, Folke Brinkmann, Sebene Mayorandan, Kristine Chobanyan-Jürgens, Anna Wiemers, Kathrin Jansen, Manfred Ballmann, Anjona Schmidt-Choudhury, Alexander Bollenbach, Nico Derichs, Dimitrios Tsikas, Thomas Lücke

**Affiliations:** 1 University Hospital of Pediatrics and Adolescent Medicine, St. Josef-Hospital, Ruhr-University Bochum, 44791 Bochum, Germany; beatrice.hanusch@rub.de (B.H.); anna.wiemers@klinikum-bochum.de (A.W.); kathrin.jansen@rub.de (K.J.); manfred.ballmann@med.uni-rostock.de (M.B.); a.schmidt-choudhury@klinikum-bochum.de (A.S.-C.); luecke.thomas@rub.de (T.L.); 2 Department of Paediatrics, Hannover Medical School, 30623 Hannover, Germany; Sebene.Mayorandan@ukmuenster.de (S.M.); Kristine.Chobanyan-Juergens@med.uni-heidelberg.de (K.C.-J.); arzt@kinderpneumologie-derichs.de (N.D.); 3 Department of Paediatrics, University Clinic Münster, 48149 Münster, Germany; 4 Department of Clinical Pharmacology and Pharmacoepidemiology, University Hospital Heidelberg, 69120 Heidelberg, Germany; 5 Department of General Pediatrics, Neuropediatrics, Metabolism, Gastroenterology, Nephrology, Center for Pediatric and Adolescent Medicine, University Hospital Heidelberg, 69120 Heidelberg, Germany; 6 Pediatric Clinical-Pharmacological Trial Center (paedKliPS), Center for Pediatric and Adolescent Medicine, University Hospital Heidelberg, 69120 Heidelberg, Germany; 7 Paediatric Clinic, University Medicine Rostock, 18057 Rostock, Germany; 8 Institute of Toxicology, Core Unit Proteomics, Hannover Medical School, 30623 Hannover, Germany; bollenbach.alexander@mh-hannover.de (A.B.); tsikas.dimitros@mh-hannover.de (D.T.); 9 KinderPneumologieDerichs, Pediatric Pneumology and Allergology, CFTR & Pulmonary Research Center, 30173 Hannover, Germany

**Keywords:** antibiotics, cystic fibrosis, inflammation, nitric oxide, *Pseudomonas aeruginosa*

## Abstract

Alterations in the L-arginine (Arg)/nitric oxide (NO) pathway have been reported in cystic fibrosis (CF; OMIM 219700) as the result of various factors including systemic and local inflammatory activity in the airways. The aim of the present study was to evaluate the Arg/NO metabolism in pediatric CF patients with special emphasis on lung impairment and antibiotic treatment. Seventy CF patients and 78 healthy controls were included in the study. CF patients (43% male, median age 11.8 years) showed moderately impaired lung functions (FEV1 90.5 ± 19.1% (mean ± SD); 21 (30%) had a chronic *Pseudomonas aeruginosa* (PSA) infection, and 24 (33%) had an acute exacerbation). Plasma, urinary, and sputum concentrations of the main Arg/NO metabolites, nitrate, nitrite, Arg, homoarginine (hArg), and asymmetric dimethylarginine (ADMA) were determined in pediatric CF patients and in healthy age-matched controls. Clinical parameters in CF patients included lung function and infection with PSA. Additionally, the Arg/NO pathway in sputum samples of five CF patients was analyzed before and after routine antibiotic therapy. CF patients with low fractionally exhaled NO (FENO) showed lower plasma Arg and nitrate concentrations. During acute exacerbation, sputum Arg and hArg levels were high and dropped after antibiotic treatment: Arg: pre-antibiotics: 4.14 nmol/25 mg sputum vs. post-antibiotics: 2.33 nmol/25 mg sputum, *p* = 0.008; hArg: pre-antibiotics: 0.042 nmol/25 mg sputum vs. post-antibiotics: 0.029 nmol/25 mg sputum, *p =* 0.035. The activated Arg/NO metabolism in stable CF patients may be a result of chronic inflammation. PSA infection did not play a major role regarding these differences. Exacerbation increased and antibiotic therapy decreased sputum Arg concentrations.

## 1. Introduction

About 1:3300 to 1:4800 newborns in Germany are affected by cystic fibrosis (CF; OMIM 219700), which is an autosomal recessively inherited disease [1]. CF is a multi-organ disease with lungs being affected most profoundly [2,3]. Chronic infection with *Pseudomonas aeruginosa* (PSA) is common in the CF population and seems to be promoted by the innate immune system response, leading to chronic inflammation in CF lungs [4]. Infection correlates with loss of lung function and reduced life expectancy [5,6,7,8,9].

L-Arginine (Arg) is a semi-essential amino acid that can be synthesized via the urea cycle [10]. It can be utilized as a substrate by nitric oxide (NO) synthase (NOS) to produce NO and L-citrulline [11,12]. NO is one of the most potent endogenous vasodilators. The Arg homolog, L-homoarginine (hArg), is produced by arginine:glycine amidinotransferase (AGAT), and it occurs at much lower concentrations in biological samples than Arg. hArg may also serve as a substrate for NOS [13]. Low circulating hArg concentrations were found to be a risk factor for all-cause mortality and cardiovascular diseases [14]. The methylated Arg analogues *N*^G^-monomethylarginine, asymmetric *N*^G^,*N*^G^-dimethylarginine (ADMA), and symmetric *N*^G^,*N^’^*^G^-dimethylarginine (SDMA) are produced by the post-translational modification of Arg residues in proteins by protein-arginine *N*-methyltransferases (PRMT), are released by proteolysis, and are inhibitors of NOS [15,16,17]. ADMA is hydrolyzed by dimethylarginine dimethylaminohydrolase (DDAH) to produce dimethylamine (DMA) and L-citrulline [18,19]. DMA in urine is a useful measure of whole-body DDAH activity [20]. The sum of urinary ADMA and DMA (i.e., ADMA + DMA) represents the whole-body asymmetric dimethylation of Arg residues in proteins. SDMA is excreted in the urine without appreciable metabolization. The sum of urinary ADMA, DMA, and SDMA (i.e., ADMA + DMA + SDMA) reflects the whole-body asymmetric and symmetric dimethylation of Arg residues in proteins [21]. In this context, the molar ratio of (ADMA + DMA)/SDMA in the urine is a measure of the ratio of asymmetric to symmetric dimethylation of proteinic Arg [21]. In addition to the Arg/NO pathway, NO can be produced in the body from inorganic nitrite and nitrate [22]. The mouth and the gut flora are predominant places of bacterial reduction of nitrate to nitrite. NO is involved in innate immune response, neurotransmission, protein function regulation, and acts as a platelet aggregation inhibitor [11,12,23].

Recently, we reported that the Arg/NO pathway is activated in pediatric CF. We found higher nitrate, nitrite, Arg, and ADMA concentrations in the plasma of pediatric CF patients compared to healthy controls as well as higher urine nitrate and DMA concentrations [24]. Changes in the Arg/NO metabolism resulting in reduced NO levels have been reported in CF patients by others as well [25,26,27,28]. Low Arg and high ADMA may contribute to airway obstruction by increasing the tone of bronchial smooth muscle. Higher sputum ADMA levels have been observed in CF patients compared to controls [25]. On the contrary, in exhaled breath condensates of pediatric CF patients, no elevation of ADMA was found. Furthermore, the Arg/ADMA ratio in CF patients with exacerbation was elevated as well [26]. Investigations of inhalative NO or Arg as treatment for lung infections showed beneficial effects [27,28].

Mucociliary clearance in bronchial and nasal epithelial cell cultures of CF patients improved even further when the arginase inhibitor CB1158 and Arg were supplemented in addition to treatment with Cystic Fibrosis Transmembrane Conductance Regulator (CFTR) modulators and potentiators (i.e., lumacaftor/ivacaftor). These effects were not seen with Arg supplementation alone [29]. Furthermore, the ingestion of nitrate-rich beetroot juice (140 mL, 12.9 mmol nitrate) or chronic doses of Arg (200 mg/kg body weight per day) for 6 weeks increased fractionally exhalative NO (FENO), which is an easily accessible non-invasive inflammatory marker, without clinical improvement [22,30].

The aim of the present study was to investigate the Arg/NO pathway in stable pediatric CF patients with special emphasis on lung impairment due to chronic and acute infection and antibiotic treatment.

## 2. Experimental Section

### 2.1. Subjects

All CF patients (*n* = 70) and healthy controls (*n* = 78) were recruited as previously reported [24], with an additional subset of each five controls and CF patients who exclusively gave sputum. The diagnosis of CF was either verified with repeated pathological sweat-tests and/or genetic analysis. CF patients under the age of five years or infected with MRSA (methicillin-resistant *Staphylococcus aureus*), *Burkholderia cepacia*, or extended-spectrum β-lactamases producing bacteria were excluded from the study. Clinical parameters were measured during routine examinations and obtained from health records. Exacerbation was defined as loss of lung function and/or increased sputum production that required antibiotic treatment [31]. Pediatric otherwise healthy controls who underwent elective control gastroscopies of asymptomatic patients at the University Children’s Hospital in Bochum or small elective surgery (e.g., circumcision or inguinal hernia surgery) at the Hannover Medical School were included into the study [24]. Written informed consent was given by parents or legal guardians. The study was approved by the Ethics Committees of the Hannover Medical School (No. 2720) and Ruhr-University Bochum (No. 3921-11). As the number of eligible data of the parameters varied due to missing or small samples in pediatric CF patients and controls, the number of CF patients and controls for each individual Arg/NO metabolite is depicted in Table S1.

### 2.2. Sampling and Biochemical Analyses

Venous blood was drawn from non-fasted children in ethylenediaminetetraacetic acid (EDTA) Monovettes (Sarstedt, Nümbrecht, Germany), put on ice, and centrifuged immediately. The plasma samples were stored at −80 °C until analysis. Spot urine samples were collected during routine check-up and stored at −80 °C until further analysis. Induced sputum of healthy controls and CF patients was collected after inhalation with salbutamol and 5.85% hypertonic saline. Some CF patients produced sputum spontaneously. Sputum was collected from five healthy untreated controls. Sputum was immediately put on ice and subsequently stored at −80 °C until analysis. After thawing on ice, portions of sputum were weighed (range, 30–240 mg) and treated under vortexing with the 4-fold volume of ice-cold acetone to precipitate proteins and inhibit enzymatic activity. After centrifugation (800× *g*, 2 °C, 5 min), 100 µL aliquots of supernatants were analyzed. For five CF patients who underwent antibiotic treatment, sputum was collected before and after two weeks of intravenous antibiotic therapy with either ceftazidime or meropenem and tobramycin. FENO was measured during yearly check-up for CF patients via NIOX^®^ (Circassia Pharmaceuticals, UK former Aerocrine, Sweden) and reported as parts per billion (ppb). Age-corrected FENO was further classified as low, normal, or high using reference FENO data [32]. Low FENO is defined as < 3rd percentile, high FENO is defined as > 95th as described before [24].

All biochemical parameters were analyzed as described elsewhere [24] by using previously reported validated methods and by considering analyte-specific features including blood and urine collection, centrifugation, sample storage, and sample work-up. Study samples were analyzed alongside quality control (QC) samples as described elsewhere for the individual analytes [33,34,35,36,37,38,39,40,41,42,43,44,45,46]. All analytes were determined with the requested accuracy (bias ≤ ± 20%) and precision (relative standard deviation, ≤20%).

Citrulline was determined using a commercially available amino acid analyzer model Biochrom 30Plus (Laborservice Onken GmbH, Gründau, Germany) based on ion-exchange high performance liquid chromatography (HPLC) and post-column derivatization with ninhydrin [38,47]. As NO production in vivo is not reliably measurable, we used nitrite and nitrate as surrogate measures of NO synthesis [39,40]. Nitrate and nitrite in plasma and urine samples were determined simultaneously by gas chromatography-mass spectrometry (GC-MS) [33,34,35]. Nitrate and nitrite in sputum were analyzed simultaneously. Arg in plasma and sputum as well as ADMA in plasma, urine, and sputum were analyzed with GC-MS/MS [37]. DMA in urine and sputum and creatinine in urine were measured by GC-MS [42,43,44]. Arg, nitrate, nitrite, and DMA were analyzed by GC-MS on an instrument model DSQ (ThermoElectron, Dreieich, Germany). ADMA was analyzed by GC-MS/MS on an instrument model DSQ (ThermoElectron, Dreieich, Germany). The urinary excretion of the NO metabolites nitrite and nitrate, of ADMA, and its metabolite DMA was corrected for creatinine excretion and reported as µM analyte per mM creatinine. Creatinine was measured by high-performance liquid chromatography [45]. Nitrate to nitrite ratios were calculated in plasma (P_NOx_R), urine (U_NOx_R), and sputum (S_NOx_R) [46]. Additionally, in sputum, hArg was measured by GC-MS on the instrument model DSQ (ThermoElectron, Dreieich, Germany). 

### 2.3. Statistical Analyses

Statistical analyses were performed using the statistical software package IBM^®^ SPSS^®^ Statistics for Windows, version 25.0 (IBM Corp., Armonk, NY, USA). Descriptive data were analyzed by the Chi-squared test or by Fisher’s exact test for groups smaller than five observations. The Kolmogorow–Smirnow test was used to test for normal distribution. Normally distributed data were analyzed using parametric tests (Student’s t-test, paired Student’s *t*-test, one-way ANOVA). Non-normally distributed data were analyzed using non-parametric tests (Mann–Whitney test, Kruskal–Wallis test). Bonferroni post hoc analysis was applied to ANOVA and Kruskal–Wallis test. Values of *p* < 0.05 were considered significant. Data are presented as mean ± standard deviation (SD) or median (25–75th interquartile range). Correlations were either performed after Pearson (*r*_p_) for normally distributed data or after Spearman (*r*_s_) for non-normally distributed data.

## 3. Results

### 3.1. Anthropometric and Clinical Characterization of CF Patients and Controls 

The CF patients recruited in the study were between 5 and 17 years old (*n* = 70, 42.9% males; median age, 11.8 years, 25–75th interquartile range 8.25–14.0 years). None of the CF patients received CFTR modulators. Healthy controls between the age of 5 and 17 years (*n* = 78, 50% males; age range, median age, 11.3 years, 25–75th interquartile range 8.19–3.2 years) were included in the study. About one-third of the CF patients (*n* = 24; 34.3%) suffered from an acute infection. About two-thirds of the CF patients (*n* = 49; 70.0%) were PSA negative. The Shwachman–Kulczycki score [48] ranked between 60 and 75. The Chrispin–Norman score [49] ranged between 0 and 20. Forced expiratory volume in 1 s (FEV1%) ranged between 36.1% and 126.1% (90.5 ± 191%). Six (8.6%) CF patients showed allergic bronchopulmonary aspergillosis (ABPA) and 14 atopy, with increased IgE levels.

### 3.2. CF vs. Control

Sputum Arg levels tended to be higher in the CF patients compared to the healthy controls (*p* = 0.052). None of the other parameters in the sputum samples differed between the groups. 

### 3.3. PSA-Positive versus PSA-Negative CF Patients 

Twenty-one CF patients were chronically infected with PSA. These patients had significantly lower FEV1% (*p* < 0.001), MEF25% (*p* < 0.001), and higher Chrispin–Norman score (*p* < 0.001) than PSA-negative patients (Table 1). No significant differences were observed in the Arg/NO pathway in plasma and urine (Table 1) or in the sputum between PSA-negative and PSA-positive patients.

### 3.4. Biochemical Parameters Grouped by FENO

**Figure 1:**
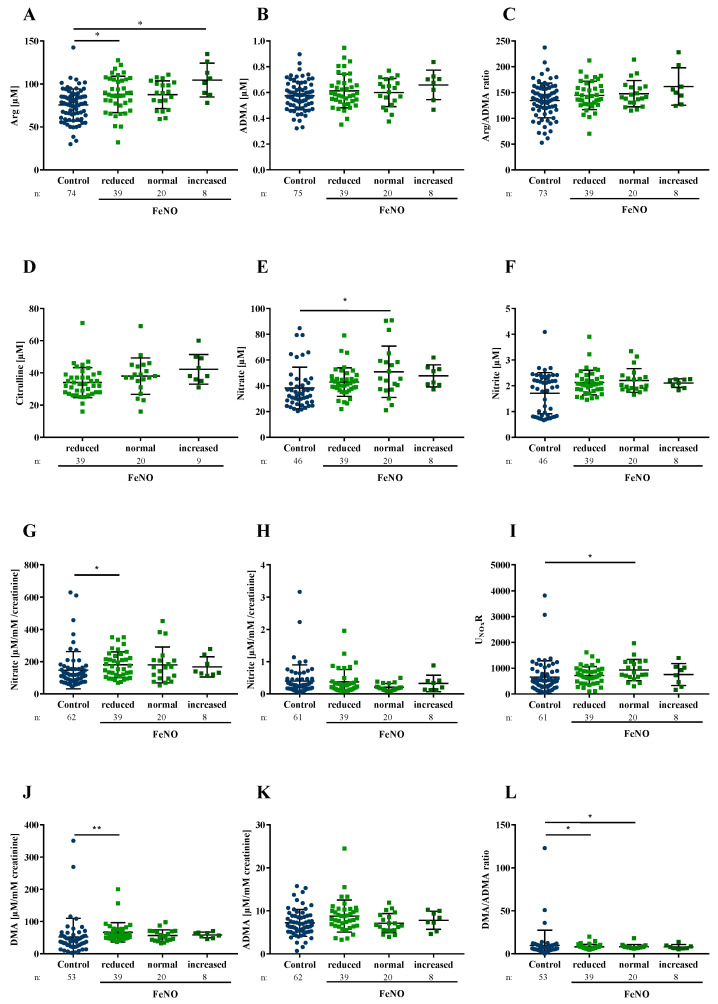
Arg/NO metabolites in urine and plasma of healthy controls and CF patients grouped by FENO. (**A**–**F**) Arg/NO metabolites in plasma; (**G**–**L**) Arg/NO metabolites in urine. (**A**) Arginine (Arg) in plasma; (**B**,**K**) Asymmetric dimethylarginine (ADMA) in plasma and urine, respectively; (**C**) Arg/ADMA ratio in plasma; (**D**) Citrulline in plasma; (**E**,**G**) Nitrate in plasma and urine, respectively; (**F**,**H**) Nitrite in plasma and urine, respectively; (**I**) Nitrate/nitrite ratio in urine (**J**), Dimethylamine (DMA) in urine; (**L**) DMA/ADMA ratio in urine. Reduced FENO < 3rd percentile, normal FENO ≥ 3rd ≤ 95th percentile, increased FENO > 95th percentile. Data were available for 68 CF patients, FENO levels were missing in one patient, Arg/NO metabolites were not available for two patients in the blood and two patients in the urine. The number of subjects varied due to missing samples and small sample volumes in pediatric CF patients and controls. * *p* < 0.05; ** *p* < 0.001

The majority (*n* = 39 (55.7%)) of CF patients had low and 9 (12.9%) high FENO levels (Figure 1). Approximately one-third of both the reduced and normal FENO groups (i.e., 13 in the reduced FENO group; 8 in the normal FENO group) were PSA positive, but none of the CF patients with increased FENO were PSA positive.

There was no difference regarding FEV1% (*p* = 0.08). However, patients with low FENO had significantly higher Chrispin–Norman scores than patients with normal FENO (*p* = 0.04).

In comparison with the healthy controls, plasma Arg was not elevated in the CF patients with normal FENO (*p* = 0.1) but significantly increased in patients with both reduced and increased FENO (reduced FENO *p* = 0.01; increased FENO *p* ≤ 0.001; Figure 1). Patients with normal FENO had higher nitrate concentrations in plasma than controls (*p* = 0.008), while the children with low or high FENO tended to also have higher plasma nitrate concentrations (reduced FENO *p* = 0.07; increased FENO *p* = 0.06, Figure 1). DMA and nitrate in urine were only significantly elevated in the reduced FENO group (DMA *p* < 0.001; nitrate *p* = 0.01, Figure 1), whereas the DMA/ADMA ratio was significantly higher in both the normal FENO group and the reduced FENO group (normal FENO *p* = 0.03; reduced FENO *p* = 0.049, Figure 1).

### 3.5. Biochemical Parameters in Sputum

Significant correlations of sputum NO parameters with the Chrispin–Norman score or FENO are summarized in Table 2. Age positively correlated with ADMA (*p* = 0.03) and Arg (*p* = 0.046) and negatively with Arg/ADMA ratio (*p* = 0.01). The Chrispin–Norman score correlated with sputum nitrite (*p* = 0.03) and Arg (*p* = 0.02). There was no correlation between sputum NO metabolites and FENO of the CF patients (Table 2).

Additionally, we tested for potential correlations between the biochemical biomarkers in plasma and urine for CF patients and healthy controls, and in sputum for CF patients. The results of these tests are summarized in Tables S2 and S3, respectively, in the Supplementary Materials. We observed multiple significant correlations. In the CF patients, nitrate and nitrite correlated strongly with each other in the sputum (*r*_p_ = 0.729, *p* = 0.026), yet not in plasma or urine. Interestingly, in the CF patients, nitrate and nitrite in sputum correlated with the nitrate/nitrite molar ratio in the urine (U_NOx_R; nitrate: *r*_p_ = 0.779, *p* = 0.013; nitrite: *r*_p_ = 0.723, *p* = 0.028), but not with the nitrate/nitrite molar ratio in the plasma (P_NOx_R).

### 3.6. Acute Infection and Inflammation

At the time of examination, 24 CF patients had an acute infection. Their lung function was significantly lower (FEV1%, *p* = 0.005; MEF25%, *p* = 0.002; Chrispin–Norman score, *p* = 0.02) in comparison to 46 CF patients without acute infection (Table 3). The BMI percentile of the acutely infected CF patients was also lower compared to the non-acutely infected CF patients (*p* = 0.02) (Table 3). CF patients with an acute infection tended to have lower plasma Arg and citrulline (*p* = 0.08 each) than CF patients without acute infection (Table 3). Furthermore, CF patients with acute infection tended to have higher sputum Arg concentrations than patients without infection (*p* = 0.08). None of the other parameters of the Arg/NO metabolism in plasma, urine, and sputum differed between the groups (Table 3).

Correlation analyses of the inflammation markers leukocyte counts and IgE with Arg/NO metabolites in plasma were conducted. Leukocyte count correlated negatively with Arg concentration (*r*_p_ = −0.342, *p* = 0.004, *n* = 68), citrulline concentration (*r*_S_ = −0.271, *p* = 0.024; *n* = 69) and the Arg/ADMA ratio (*r*_P_ = −0.328, *p* = 0.006, *n* = 68) Median IgE levels in CF patients were 60.5 IE/mL (25–75th percentile: 12.0–256.7 IE/mL). In the group with high FENO, IgE levels did not differ compared to the normal and reduced FENO group (*p* = 0.486). Of these nine patients with high FENO, none had ABPA and one atopy.

### 3.7. Antibiotic Treatment

**Figure 2:**
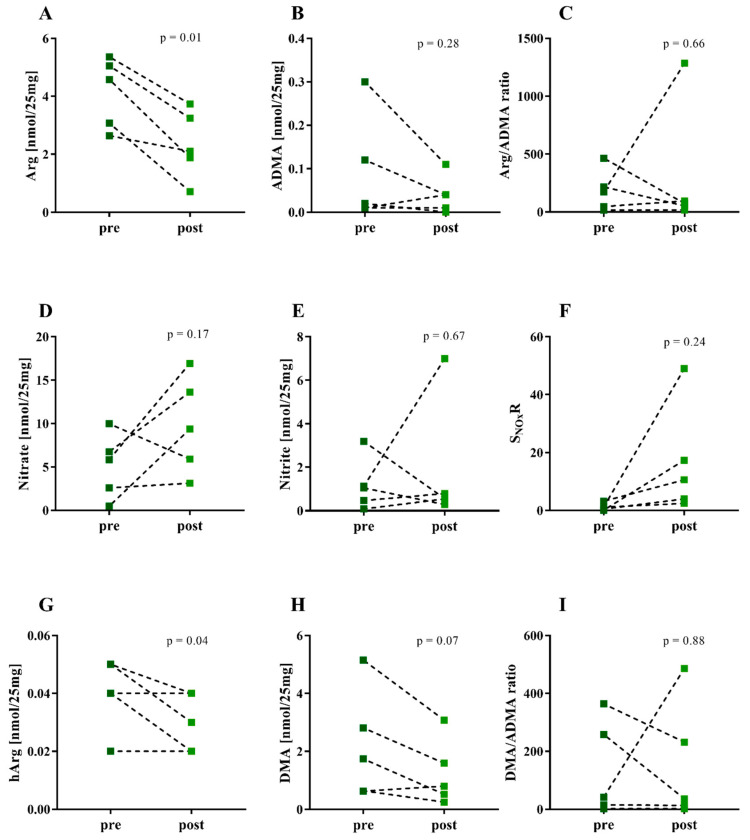
Content (nmol analyte per 25 mg sputum) of the indicated Arg/NO metabolites in sputum samples of five CF patients before and after antibiotics intravenous infusion. (**A**) Arg, arginine; (**B**) ADMA, asymmetric dimethylarginine; (**C**) Arg/ADMA ratio, (**D**) nitrate; (**E**) nitrite; (**F**) S_NOx_R nitrate/nitrite ratio in sputum, (**G**) hArg, homoarginine; (**H**) DMA, dimethylamine; (**I**) DMA/ADMA ratio.

Five CF patients were treated with antibiotics. Sputum was collected before treatment and after two weeks of intravenous antibiotic therapy. The results are illustrated in Figure 2. In the CF patients, the Arg (*p* = 0.01) and hArg (*p* = 0.04) were significantly reduced after antibiotic treatment, and DMA tended to be reduced (*p* = 0.07) after infusion (Figure 2). The ADMA, nitrate, and nitrite content of the sputum did not change upon antibiotic treatment.

### 3.8. One-Year Changes in the Arg/NO Pathway in Plasma and Urine of Pediatric Cystic Fibrosis Patients

The anthropometric, clinical, and biochemical parameters, including FENO values and the plasma and urinary concentrations of metabolites of the Arg/NO pathway, were measured in ten CF patients (six females, four males; age range, 6.0–15.0 years; *n* = 10, F508del; *n* = 1, R553) at two different time points with a between interval of one year (1.0 ± 0.1 years). The results of these analyses are summarized in Table 4.

Mean FENO increased from 7.1 to 10.3 (+45%), but the increase failed statistical significance (*p* = 0.077). Among the plasma parameters, the mean concentration of nitrite tended to increase after one year by about 31%. In the urine, the strongest changes (−31%) were seen in DMA excretion, yet decreases in DMA and in its composites did not reach statistical significance (DMA/ADMA ratio *p* = 0.071, ADMA+DMA *p* = 0.065, (ADMA+DMA)/SDMA *p* = 0.076; Table 4).

We tested for correlations after Spearman between FENO values and plasma or urinary Arg/NO biomarker concentrations at two time points, year zero and one year later. At year zero, FENO correlated with plasma Arg (*r*_S_ = 0.64, *p* = 0.05), plasma hArg (*r*_S_ = 0.60, *p* = 0.07), and U_NOx_R (*r*_S_ = 0.79, *p* = 0.01). Inverse correlations were observed between FENO and DMA (*r*_S_ = −0.80, *p* = 0.01), ADMA+DMA (*r*_S_ = −0.78, *p* = 0.02), ADMA+DMA+SDMA (*r*_S_ = −0.73, *p* = 0.03), nitrate (*r*_S_ = −0.65, *p* = 0.05), or nitrite (*r*_S_ = −0.85, *p* = 0.01). At one year, FENO correlated reversely only with urinary nitrate (*r*_S_ = −0.70, *p* = 0.03), whereas the correlations with DMA (*r*_S_ = −0.59, *p* = 0.08) and (ADMA+DMA)/SDMA (*r*_S_ = −0.61, *p* = 0.73) failed statistical significance.

## 4. Discussion

Progressive pulmonary impairment is the most relevant prognostic factor regarding life expectancy in CF patients. Chronic infection with PSA is common in CF and leads to chronic inflammation [4] and subsequent loss of lung function [5,6,7,8,9]. Previously, we investigated the status of the Arg/NO pathway in CF and healthy children and found elevated concentrations of major metabolites of the Arg/NO pathway in plasma and urine suggesting a generally activated metabolic system including high systemic and whole-body NO synthesis [24].

The aim of the present study was to investigate a potential role of the pulmonary Arg/NO system in the development, progression, and possibly in the treatment of pediatric CF lung disease [2,3]. Therefore, we assessed the Arg/NO pathway in sputum samples of children with CF and healthy children. In CF patients, different clinical conditions, including progression of the disease, PSA infection, and antibiotic therapy as well as relevant clinical parameters such as FENO were assessed and correlated with parameters of the Arg/NO pathway in sputum, plasma, and urine. The major findings of the present study are the following. (1) The activated Arg/NO pathway in plasma and urine of stable pediatric CF patients compared to healthy controls [24] was not influenced by the extent of CF lung disease. (2) Sputum Arg and sputum nitrite correlated positively with Chrispin–Norman score, which is a marker for disease severity. (3) Routine antibiotic therapy for 14 days decreased the concentrations of Arg and hArg in the sputum, whereas neither chronic nor acute PSA infection significantly influenced the Arg/NO pathway in sputum, plasma, and urine.

### 4.1. Effects of Chronic PSA Infection in Pediatric CF Patients

Chronic PSA infection is one of the risk factors for the progression of CF lung disease. In our cohort, CF patients with chronic PSA infection had significantly lower FEV1 and higher Chrispin–Norman scores compared to the CF patients without PSA infection. FEV1 is a routinely used marker of lung function, declining with lung impairment [50], whereas the Chrispin–Norman score evaluates radiologic changes in lungs and therefore rises with increasing lung destruction [49]. The FENO is widely used to assess non-invasively airway inflammation in various diseases including asthma and chronic obstructive pulmonary disease (COPD) in clinical practice [51]. The utility of FENO as a biomarker of chronic airway inflammation is based on the assumption that the inducible NOS form (iNOS) in the airway epithelium contributes to exhaled NO (reviewed in Ref. [51]). There is indeed evidence from a clinical study in healthy (baseline FENO, 6 ppb) and asthmatic (FENO, 30 ppb) humans that iNOS is the main contributor to FENO [52]. To investigate chronic inflammation by PSA in the airways, we measured FENO levels in our CF patients as well as the status of Arg/NO pathway. In our study, we did not observe differences in FENO and markers of Arg/NO metabolism in blood and urine between PSA-infected and PSA-non-infected CF children. This observation may suggest that FENO does not depend upon PSA infection in pediatric CF. Chronically PSA infected CF patients are routinely treated with the inhaled antibiotic tobramycin. Barsoumian et al. observed lower NO production in mice treated systemically with tobramycin compared to mice treated with saline [53]. We cannot exclude a possible effect of inhaled tobramycin on systemic NO production. Therefore, this might be an interesting field of future research [53].

In healthy subjects, a homeostasis of arginase and NOS activity is presumed [54]. Arginase is a widely distributed very active enzyme that hydrolyzes L-arginine to L-ornithine and urea [55,56,57]. Elevated arginase activity might result from lung inflammation due to infection. Arginase is commonly released during inflammation, cell damage, and death, and by certain bacteria [10,25,54,58,59]. Elevated arginase expression/activity may result in a decrease of Arg bioavailability. As Arg is the substrate of all known NOS isoforms, competition for their common substrate Arg by NOS and arginase may lead to diminished NO synthesis throughout the body [55,56,57], as well as in the lungs. In the latter case, this may reduce FENO levels in CF [60]. However, we observed even higher Arg concentrations both in the plasma and in the sputum of our pediatric CF patients compared to healthy children [24]. Moreover, both Arg and FENO levels did not differ between PSA-infected and PSA-non-infected CF children. These observations suggest that arginase did not play a significant role in the pediatric CF patients of our study. Our results are supported by findings from others who found no differences in any of the NO parameters in the lung, nose, and blood of 38 CF patients with regard to PSA infection [61]. Nitrate and nitrite levels were elevated in PSA-infected mice lungs and increased with arginase inhibitor application [62]. PSA is able to use denitrification pathways to grow. Therefore, the sputum of CF patients, which is rich in nitrogen products due to chronic lung inflammation [63], might serve as ideal media for PSA.

Additionally, Arg promotes PSA biofilm production and slows down swarming motility [64]. Biofilm production protects PSA bacteria from being attacked by the patients’ own immune system and antibiotics [65]. Consecutively, high Arg plasma levels might be a cause for higher PSA susceptibility for this pathogen. In our previous paper, we showed an accelerated whole body NO synthesis in pediatric CF patients, assessed by higher nitrate and nitrite plasma concentrations [24], which was not further elevated by PSA infection, as shown here. Therefore, chronically high plasma Arg concentrations seen in pediatric CF patients are likely to stem from chronic systemic inflammation rather than from the inflamed lungs.

As the activity of the immune system might contribute to NO production and therefore influence FENO [11,12,23], leukocyte count and IgE (a marker of allergic inflammation) are also assessed. In adults with chronic cough and pediatric asthmatics, a higher FENO correlated with higher blood eosinophilic counts [66,67]. No such correlation was seen in CF patients, but a negative correlation with neutrophils count was observed [61]. Therefore, FENO levels in CF are affected by infection rather than by allergic inflammation [61]. Accordingly, IgE did not correlate with FENO in our CF patients either, indicating that allergic inflammation in the airways of our patients does not play a significant role. Interestingly, leukocyte count correlated negatively with Arg, citrulline, and Arg/ADMA ratio in plasma, indicating a reduced loss of NO with less inflammatory activity and therefore higher residual Arg, citrulline, and Arg/ADMA levels in plasma. As less NO is lost by reactions with other reactive oxygen species produced during inflammatory processes, NOS is likely to consume less Arg to produce NO and citrulline.

### 4.2. Systemic Differences in CF Groups Regarding FENO

We divided our CF patients according to their FENO levels into low FENO, normal FENO, and high FENO groups. We observed higher Chrispin–Norman scores and slightly lower FEV1 levels in the low FENO group, but no significant differences in plasma and urinary Arg/NO metabolism markers between the CF patients. In infants with CF, lower FENO levels were measured than in healthy infants (12.2 (10.2–16.3) ppb vs. 16.4 (13.8–18.7) ppb, *p* < 0.001) [68]. Lower FENO levels in CF patients compared to healthy controls have been also reported by others, for instance by Winter-de Groot and van der Ent [69], by Robroeks et al. (10 vs. 15.3 ppb, *p* < 0.05) [70], and Keen et al. (9.5 (2.7–33.8) vs. 12.4 (5.2–40.1), *p* = 0.029) [71]. Interestingly, in asthmatic and COPD patients, higher FENO levels were found during exacerbation [66,72,73].

Based on these data, it can be concluded that changes in FENO values are associated with disease in CF, asthma, and COPD, and that iNOS is its main contributor, but PSA infection does not influence these changes further. 

iNOS is little expressed in epithelial cells of CF patients’ lungs, without a significant decline of NO production in CF lung tissue [74]. As stable pediatric CF patients show in general higher Arg, nitrate, and nitrite plasma concentrations compared to controls, NO production capacity is not reduced in pediatric CF patients [24]. An augmented occurrence of reactive oxygen species also leads to an accelerated NO depletion by superoxide anion reacting with NO to form peroxynitrite [69,75]. Therefore, reduced FENO levels probably develop due to faster clearance of the NO radical rather than by diminished production. This might explain the comparable plasma nitrate and nitrite levels in the different CF groups. 

In our 1-year follow-up study, median FENO tended to increase from 7.1 to 10.3 (*p* = 0.077). Factors associated with changes in health-related quality of life (HRQoL) in 39 pediatric CF patients (age range, 6–18 years) were investigated during 1-year follow-up [76]. HRQoL was found to significantly improve, yet without changes in FEV1% (*p* = 0.987) [76]. This observation is in line with our finding in the present study on 10 CF children (*p* = 0.573).

### 4.3. Effects of Acute Infection in Pediatric CF Patients

As chronic inflammation of the lungs by PSA did not show an impact on Arg/NO metabolism, patients with an acute infection were analyzed further. Pediatric CF patients with acute infection showed reduced lung function and lower Arg as well as citrulline concentrations in plasma compared to CF patients without acute infection. This indicates lower Arg availability for NOS as described previously by Grasemann et al. [28]. We found a tendency of reduced plasma Arg and citrulline in CF patients during acute infection compared to CF patients without acute infection. No changes of ADMA plasma concentration and Arg/ADMA ratio were observed with acute infection. However, these lower Arg plasma concentrations in CF patients with acute infection still stayed above levels seen in healthy controls as described before [24]. These observed changes might be due to Arg consumption by the patient’s immune system and microbiome. As plasma Arg concentration does not depend on age, plasma concentrations of Arg are comparable in children and adults and do not need age-correction [17,77].

Stable CF patients seem to show elevated plasma Arg levels, which fall during exacerbation. The plasma Arg/ADMA ratio was significantly higher in our stable pediatric CF patients than in controls [24], and it was positively correlated with improved lung function parameters. CF patients showing an acute infection had significantly worse lung function and tended to have lower plasma citrulline and Arg and higher sputum Arg. These observations correlate with breath condensate analyses by Lucca et al. in pediatric CF patients [26]. As we found no differences of ADMA in CF patients with acute infection, ADMA clearance might not be impaired in these patients. NOS activity is not only regulated by ADMA inhibition but also by cellular Arg uptake. Furthermore, we observed increased sputum Arg in patients with acute infection. Higher Arg concentration in the sputum during acute infection may be explained by a varied distribution of Arg in lungs and systemically. Since stable pediatric CF patients show an increased systemic Arg/NO metabolism [24], a further increase of Arg could be an excessive attempt to further activate NO synthesis during acute inflammation. Since the PSA infection often becomes chronic, a further increase of Arg does not occur here, but it is lost in the underlying inflammatory type of CF patients.

### 4.4. Effects of Antibiotic Treatment in Pediatric CF Patients

Grasemann et al. found low Arg in CF patients that were normalized after 14 days of antibiotic treatment [77]. Engelen et al. also observed higher Arg plasma concentrations after antibiotic treatment in pediatric CF patients compared to controls [78].

Accordingly, one could state that stable CF patients seem to show elevated plasma Arg levels, which fall during exacerbation and recover after treatment. This was also investigated in the sputum of five CF patients, who underwent routine antibiotic treatment, before and after 14 days of treatment. The concentrations of Arg, hArg, and DMA and in the sputum of these CF patients were lower after the 14 days of antibiotic treatment. As much less than 0.1% of body Arg is utilized in the Arg/NO pathway and nitrate and nitrite in sputum did not change by antibiotic treatment, other Arg-involving mammalian and bacterial pathways with higher Arg utilization are likely to have been involved. These pathways may include protein synthesis and AGAT-catalyzed conversion of Arg and lysine to guanidinoacetate and hArg [13,79].

As Arg is part of creatine synthesis, higher demands of creatine could require higher amounts of Arg in lungs, leading to a shift from plasma to lungs. Braegger et al. analyzed creatine kinase activity in nasal, tracheal, and bronchial epithelia. The creatine kinase showed low activity, which was not increased after creatine supplementation, nor was the lung function in CF patients supplemented with creatine [80]. 

Another explanation for elevated Arg sputum levels in CF patients prior to antibiotic treatment could be microbial Arg synthesis and secretion. Indeed, certain bacteria have been found to excrete Arg and L-lysine [79]. As Arg and L-lysine are used by AGAT to produce hArg [13], the higher hArg levels in the sputum of our CF patients may be due to increased AGAT activity in the sputum. It is worth mentioning that in vitro Arg enhances significantly the antibiotic susceptibility of PSA, while Arg alone showed no effect on PSA growth [81]. Therefore, elevated sputum Arg concentrations could be used to assess the monitoring of (intravenous) antibiotic therapy with regard to duration and choice of drugs. 

We did not observe appreciable changes in the Arg/NO pathway changes and in clinical parameters in the 1-year follow up study in ten of our CF patients, indicating clinically stable CF patients. We observed minor changes in whole-body asymmetric and symmetric Arg dimethylation in proteins [21].

### 4.5. Study Limitations

The pediatric CF patients investigated in the present study were in good clinical condition. Only two patients had FEV1% below 50%. A possible limitation of our study is that children were not fasted overnight and were not required to refrain from foods rich in citrulline, Arg, nitrate, and nitrite prior to blood, urine, and sputum sampling. Yet, this limitation applies both to the CF patients and to the healthy controls, thus possibly balancing the contribution of exogenous Arg and its metabolites to the analytes measured in the study samples. Another limitation of our study is the relatively small number of CF patients and controls who donated the sputum samples and the small number of CF patients who received intravenously antibiotics. 

## 5. Conclusions

In our stable pediatric CF patients, the Arg/NO pathway is upregulated independent of PSA infection. Our pediatric CF patients are sufficiently supplied by Arg, as seen in high plasma Arg levels that do not show signs of deficiency. The chronically activated Arg/NO pathway in pediatric CF may be due to chronic inflammation by other unknown factors in the lung or other organs such as the liver, pancreas, or the gut. The sputum of CF children contains higher amounts of Arg and hArg than the sputum of healthy children. Standard antibiotic treatment decreases the sputum content of Arg and hArg, which is associated with a small increase in FENO levels. This is the first study to demonstrate elevated hArg levels in the sputum of pediatric CF patients and their decrease by intravenously administered antibiotics.

## Figures and Tables

**Table 1:** Comparison between cystic fibrosis (CF) patients without and with *Pseudomonas aeruginosa* (PSA) infection.

	PSA-Negative CF	PSA-Positive CF	*p*-Value
	49	21	-
Age (years)	11.8 (8.1–13.9)	11.1 (8.6–14.6)	0.654
Male (*n* (%))	20 (40.8)	10 (47.6)	0.598
Shwachman–Kulczycki score	75.0 (70.0–75.0)	72.5 (70.0–75.0)	0.076
**FEV1 (%)**	**97.4 ± 16.4**	**74.3 ± 14.9**	**0.000**
**MEF25 (%)**	**63.0 (47.2–91.5)**	**41.7 ± 25.1**	**0.000**
**Chrispin–Norman score**	**4.0 (2.0–6.0)**	**9.0 (4.5–14.5)**	**0.000**
FENO (ppb)	8.80 (6.13–13.53)	7.70 (4.75–11.85)	0.192
GFR (mL/min)	137 ± 19.9	142 ± 20.2	0.305
Plasma			
Arg (µM)	93.0 ± 20.9	84.3 ± 18.6	0.107
Citrulline (µM)	36.5 (29.3–44.0)	35.0 (28.5–39.0)	0.337
ADMA (µM)	0.63 ± 0.12	0.59 ± 0.14	0.295
Arg/ADMA	149 ± 29	144 ± 25	0.508
Nitrate (µM)	42.5 (37.4–51.1)	44.1 (37.7–58.9)	0.478
Nitrite (µM)	2.06 (1.85–2.26)	2.10 (1.87–2.47)	0.360
P_NOx_R	21.8 (17.3–25.3)	20.3 (17.2–22.8)	0.600
Urine			
ADMA (µM/mM creatinine)	7.58 (6.30–9.98)	7.87 (6.16–9.86)	0.789
DMA (µM/mM creatinine)	58.3 (46.8–70.5)	56.5 (47.1–70.63	0.755
DMA/ADMA	7.69 (6.41–9.00)	7.43 (6.35–7.95)	0.637
Nitrate (µM/mM creatinine)	141 (113–210)	208 (96–264)	0.174
Nitrite (µM/mM creatinine)	0.20 (0.12–0.33)	0.28 (0.15–0.50)	0.329
U_NOx_R	746 (462–1024)	746 (541–1058)	0.963

Abbreviations: ADMA, asymmetrical dimethylarginine; Arg, Arginine; Arg/ADMA, Arg/ADMA ratio; DMA, dimethylamine; DMA/ADMA, dimethylamine/asymmetrical dimethylarginine ratio; FENO, fractionally exhaled nitric oxide; FEV1, Forced expiratory volume in 1 s; GFR = glomerular filtration rate; MEF25, Maximal expiratory flow 25%; P_NOx_R, nitrate/nitrite ratio in plasma; ppb = parts per billion; U_NOx_R, nitrate/nitrite ratio in urine. Data are reported as median (25–75 percentile) (non-normal distribution) or as mean ± standard deviation (normal distribution). Significant results are marked bold.

**Table 2:** Pearson correlation coefficients (*r*_P_) and statistical significance (*P* value) between metabolites of the Arg/NO pathway in sputum of cystic fibrosis patients (*n* = 9) and age, Chrispin–Norman score, or FENO.

Parameter	Age (*y*)		Chrispin-Norman Score		FENO (ppb)	
	*r*_P_	*p*	*r*_P_	*p*	*r*_P_	*p*
Arg	**0.676**	**0.046**	**0.736**	**0.024**	−0.534	0.138
ADMA	**0.707**	**0.033**	0.546	0.128	−0.229	0.553
Arg/ADMA	**−0.782**	**0.013**	−0.248	0.520	0.136	0.727
hArg	0.307	0.422	0.109	0.781	−0.194	0.617
DMA	−0.187	0.631	−0.173	0.656	−0.247	0.521
DMA/ADMA	−0.315	0.409	−0.25	0.517	−0.130	0.739
Nitrate	0.168	0.665	0.414	0.268	0.309	0.418
Nitrite	0.436	0.241	**0.728**	**0.026**	−0.081	0.836
S_NOx_R	−0.274	0.476	−0.476	0.196	0.490	0.181

Abbreviations: Arg, arginine; ADMA, asymmetric dimethylarginine; Arg/ADMA, arginine/asymmetric dimethylarginine ratio; DMA, dimethylamine; DMA/ADMA, dimethylamine/asymmetric dimethylarginine ratio; FENO, fractionally exhaled NO; hArg, homoarginine; ppb, parts per billion; S_NOx_R, nitrate/nitrite ratio in sputum. Concentrations of all parameters were nmol of analyte per 25 mg of sputum. Significant results are marked in bold.

**Table 3:** Comparison of anthropometric, clinical, and biochemical parameters of cystic fibrosis patients without and with acute infection.

Anthropometric and Clinical Parameter	No Acute Infection (*n* = 46)	Acute Infection (*n* = 24)	*p*-Value
Age (years)	11.4 (8.2–14.0)	12.41 (8.4–14.2)	0.757
**Male (*n* (%))**	**24 (52.2)**	**6 (25.0)**	**0.042**
**BMIp (percentile)**	**47.5 ± 27.9**	**30.9 ± 24.8**	**0.016**
**Shwachman–Kulczycki score**	**75 (75–75)**	**70 (70–75)**	**0.001**
**FEV1 (%)**	**95.0 ± 18.1**	**81.7 ± 18.3**	**0.005**
**MEF25 (%)**	**63.0 (52.3–87.8)**	**39.5 (26.5–53.9)**	**0.002**
**Chrispin-Norman score**	**4.0 (2.00–6.5)**	**8.00 (4.0–10.0)**	**0.021**
FENO (ppb)	8.3 (6.0–12.7)	8.4 (4.9–13.5)	0.935
GFR (mL/min)	138.1 ± 18.2	140.0 ± 23.5	0.705
Plasma			
Arg (µM)	93.5 ± 19.2	84.2 ± 21.9	0.077
Citrulline (µM)	37.0 (31.5–44.3)	33.0 (28.0–39.0)	0.078
ADMA (µM)	0.63 ± 0.12	0.59 ± 0.12	0.203
Arg/ADMA	149.5 ± 25.2	144.0 ± 32.9	0.446
Nitrate (µM)	43.0 (38.0–52.3)	44.1 (36.5–52.0)	0.821
Nitrite (µM)	2.08 (1.86–2.27)	2.03 (1.86–2.27)	0.938
P_NOx_R	21.4 (17.5–25.5)	20.5 (16.7–23.2)	0.315
Urine			
ADMA (µM/mM creatinine)	8.37 ± 3.54	7.80 ± 2.51	0.485
DMA (µM/mM creatinine)	58.8 (49.3–70.2)	53.9 (44.3–71.1)	0.317
DMA/ADMA	7.67 (6.42–8.87)	7.10 (6.13–8.29)	0.538
Nitrate (µM/mM creatinine)	159 (11.6–215)	159 (114–246)	0.898
Nitrite (µM/mM creatinine)	0.20 (0.12–0.38)	0.22 (0.16–0.33)	0.542
U_NOx_R	838 ± 403	6945 ± 325	0.138
Sputum			
Arg (nmol/25 mg)	2.22 ± 1.03	4.18 ± 1.69	0.083^a^
ADMA (nmol/25 mg)	0.01 ± 0.01	0.03 ± 0.03	0.133^a^
Arg/ADMA	262 ± 131	153 ± 48	0.197^a#^
hArg (nmol/25 mg)	0.04 ± 0.004	0.04 ± 0.003	0.448^a^
DMA (nmol/25 mg)	0.28 ± 0.18	1.71 ± 2.92	0.368^a^
DMA/ADMA	55.5 ± 78.4	145 ± 304	0.587^a^
Nitrate (nmol/25 mg)	18.3 ± 1.79	10.9 ± 9.56	0.175^a^
Nitrite (nmol/25 mg)	2.60 ± 1.51	3.25 ± 3.16	0.719^a^
S_NOx_R	9.03 ± 4.84	4.34 ± 2.78	0.109^a^

Abbreviations. BMI, body mass index; FEV1, forced expiratory volume in 1 s; MEF25, maximal expiratory flow 25%; FENO, fractionally exhaled nitric oxide; GFR, glomerular filtration rate; Arg, Arginine; ADMA, asymmetrical dimethylamine; Arg/ADMA, arginine/asymmetric dimethylarginine ratio; hArg, homoarginine; DMA, dimethylamine; DMA/ADMA, dimethylamine/asymmetric dimethylarginine ratio; P_NOx_R, nitrate/nitrite ratio in plasma; ppb, parts per billion; S_NOx_R, nitrate/nitrite ratio in sputum; U_NOx_R, nitrate/nitrite ratio in urine. Data are reported as median (25–75th percentile) (non-normal distribution) and as mean ± standard deviation (normal distribution). Significant results are marked in bold. ^#^ heteroscedasticity; ^a^ sample size, *n* = 4 for no infection, and *n* = 5 for acute infection.

**Table 4:** One-year follow-up of clinical parameters and plasma and creatinine-corrected urinary concentrations of main metabolites of the Arg/NO pathway in ten cystic fibrosis patients.

Clinical Parameters	Start	One Year	*p*-Value
FENO (ppb)	7.1 ± 4.1	10.3 ± 5.3	0.077
Shwachman–Kulczycki score	72.5 ± 3.5	72.5 ± 4.2	>0.999
Chrispin–Norman score	5.9 ± 3.9	6.0 ± 3.5	0.859
FEV1 (%)	94.3 ± 15.4	96.2 ± 16.4	0.573
MEF25 (%)	76.3 ± 58.2	78.0 ± 45.7	0.892
Plasma			
Arg (µM)	79.8 ± 27.4	91.7 ± 26.4	0.315
hArg (µM)	2.01 (0.69–4.32)	2.27 (1.43–4.93)	0.922
Citrulline	32.6 ± 7.0	32.2 ± 8.1	0.870
ADMA (µM)	0.57 ± 0.15	0.54 ± 0.08	0.519
Nitrate (µM)	43.7 ± 10.0	43.2 ± 12.4	0.919
Nitrite (µM)	1.9 ± 0.4	2.5 ± 0.6	0.071
P_NOx_R	24.3 ± 9.7	18.2 ± 6.2	0.121
Urine			
ADMA (µM/mM)	8.5 ± 6.1	6.9 ± 1.9	0.414
DMA (µM/mM)	76.1 ± 46.0	52.7 ± 8.0	0.125
DMA/ADMA	9.9 ± 3.7	7.9±1.6	0.071
SDMA (µM/mM)	7.77 (5.80–8.78)	8.02 (6.89–9.04)	0.376
ADMA+DMA	73.2 (55.4–86.0)	62.8 (51.9–65)	0.065
ADMA+DMA+SDMA	80.5 (60.7–86.6)	71.4 (57.6–74.5)	0.129
(ADMA+DMA)/SDMA	8.33 (8.1–10.8)	7.61 (6.68–8.99)	0.076
Nitrate (µM/mM)	163.8 ± 83.6	145.1 ± 55.7	0.353
Nitrite (µM/mM)	0.30 ± 0.29	0.25 ± 0.16	0.490
U_NOx_R	770 ± 319	717 ± 341	0.613

Abbreviations: FENO, fractionally exhaled nitric oxide; FEV1, Forced expiratory volume in 1 s; MEF25, Maximal expiratory flow 25%; Arg, arginine; hArg, homoarginine; ADMA, asymmetrical dimethylamine; Arg/ADMA, arginine/asymmetric dimethylarginine ratio; DMA, dimethylamine; DMA/ADMA, dimethylamine/asymmetric dimethylarginine ratio; P_NOx_R, nitrate/nitrite ratio in plasma; ppb, parts per billion; S_NOx_R, nitrate/nitrite ratio in sputum; U_NOx_R, nitrate/nitrite ratio in urine. Data are reported as median (25–75th percentile) (non-normal distribution) and as mean ± standard deviation (normal distribution).

## Supplementary Materials

The following are available online at https://www.mdpi.com/2077-0383/9/12/3802/s1. Table S1. Number of CF patients and controls with eligible data for the mentioned Arg/NO parameters. Table S2. Summary of the significant results of Pearson correlation analyses between members of the Arg/NO pathway in sputum (S), plasma (P) and urine (U) in cystic fibrosis patients who gave sputum. Table S3. Summary of the results of correlation analyses between members of the Arg/NO pathway in plasma (P) and urine (U) in the controls of the study.
