# Supplementary material for: Local and Systemic Alterations of the L-Arginine/Nitric Oxide Pathway in Sputum, Blood, and Urine of Pediatric Cystic Fibrosis Patients and Effects of Antibiotic Treatment
Source: J Clin Med. 2020 Nov 24;9(12):3802. doi: 10.3390/jcm9123802 (PMC7761143; doi:10.3390/jcm9123802)
Supplement: Supplementary file 1 [file jcm-09-03802-s001.pdf]

Journal of
Clinical Medicine

MDPI

Supplement

Local and Systemic Alterations of the L-Arginine/Nitric Oxide Pathway in the Sputum, Blood and Urine of Pediatric Cystic Fibrosis Patients and Effects of Antibiotic Treatment

Beatrice Hanusch [1,†], Folke Brinkmann [1,*,†], Sebene Mayorandan [2,3], Kristine Chobanyan-Jürgens [2,4,5,6], Anna Wiemers [1], Kathrin Jansen [1], Manfred Ballmann [1,7], Anjona Schmidt-Choudhury [1], Alexander Bollenbach [8], Nico Derichs [2,9],Dimitrios Tsikas [8,‡] and Thomas Lücke [1,‡]

1 University Hospital of Pediatrics and Adolescent Medicine, St. Josef-Hospital, Ruhr-University Bochum, 44791 Bochum, Germany; beatrice.hanusch@rub.de (B.H.); anna.wiemers@klinikum-bochum.de (A.W.); kathrin.jansen@rub.de (K.J.); manfred.ballmann@med.uni-rostock.de (M.B.); a.schmidt-choudhury@klinikum-bochum.de (A.S.-C.); luecke.thomas@rub.de (T.L.)
2 Department of Paediatrics, Hannover Medical School, 30623 Hannover, Germany; Sebene.Mayorandan@ukmuenster.de (S.M.); Kristine.Chobanyan-Juergens@med.uni-heidelberg.de (K.C.-J.); arzt@kinderpneumologie-derichs.de (N.D.)
3 Department of Paediatrics, University Clinic Münster, 48149 Münster, Germany
4 Department of Clinical Pharmacology and Pharmacoepidemiology, University Hospital Heidelberg , 69120 H eidelberg, Germany
5 Department of General Pediatrics, Neuropediatrics, Metabolism, Gastroenterology, Nephrology, Center for Pediatric and Adolescent Medicine, University Hospital Heidelberg, 69120 Heidelberg, Germany
6 Pediatric Clinical-Pharmacological Trial Center (paedKliPS), Center for Pediatric and Adolescent Medicine, University Hospital Heidelberg, 69120 Heidelberg, Germany
7 Paediatric Clinic, University Medicine Rostock, 18057 Rostock, Germany
8 Institute of Toxicology, Core Unit Proteomics, Hannover Medical School, 30623 Hannover, Germany; bollenbach.alexander@mh-hannover.de (A.B.); tsikas.dimitros@mh-hannover.de (D.T.)
9 KinderPneumologieDerichs, Pediatric Pneumology and Allergology, CFTR & Pulmonary Research Center, 30173 Hannover, Germany
* Correspondence: folke.brinkmann@rub.de; Tel.: +49-234-5092680
† Shared first authors.
‡ Shared senior authors.

Received: date; Accepted: date; Published: 24 November 2020

Table S1: Number of CF patients and controls with eligible data for the mentioned Arg/NO parameters.

	Number of CF patients	Number of healthy controls
Arg (P)	68	74
Citrulline (P)	69	0
ADMA (P)	68	75
Arg/ADMA (P)	68	73
Nitrate (P)	68	46
Nitrite (P)	68	46
$P_{NOx}R$	68	46
ADMA (U)	68	62
DMA (U)	68	53
DMA/ADMA (U)	68	53
Nitrate (U)	68	62
Nitrite (U)	68	61
$U_{NOx}R$	68	61

Abbreviations. Arg, Arginine; ADMA, asymmetric dimethylamine; Arg/ADMA, arginine/asymmetric dimethylarginine ratio; DMA, dimethylamine; DMA/ADMA, dimethylamine/asymmetric dimethylarginine ratio; (P), plasma; $P_{NOx}R$, nitrate/nitrite ratio in plasma; (U), urine; $U_{NOx}R$, nitrate/nitrite ratio in urine. The number of subjects varied due to missing samples and small sample volumes in pediatric CF patients and controls.

Table S2. Summary of the significant results of Pearson correlation analyses between members of the Arg/NO pathway in sputum (S), plasma (P) and urine (U) in cystic fibrosis patients who gave sputum.

Correlation pair	Correlation coefficient	*P* value	Data pairs (*n*)
Arg (S) vs ADMA (S)	0.896	0.001	9
ADMA (S) vs Arg/ADMA (S)	-0.673	0.047	9
Nitrate (S) vs Nitrite (S)	0.729	0.026	9
DMA (S) vs DMA/ADMA (S)	0.973	< 0.001	9
Arg/ADMA(S) vs ADMA (U)	0.719	0.029	9
Arg/ADMA(S) vs Nitrate (U)	0.747	0.021	9
Nitrate (S) vs $U_{NOx}R$	0.779	0.013	9
Nitrite (S) vs $U_{NOx}R$	0.723	0.028	9
$S_{NOx}R$ vs DMA/ADMA (U)	0.693	0.038	9
Arg (P) vs Arg/ADMA (P)	0.780	0.013	9
ADMA (P) vs Nitrite (P)	-0.796	0.010	9
ADMA (P) vs $P_{NOx}R$	0.710	0.032	9
Nitrate (P) vs Arg/ADMA (P)	-0.736	0.024	9
Nitrate (P) vs $P_{NOx}R$	0.937	< 0.001	9
ADMA (P) vs ADMA (U)	0.678	0.045	9
ADMA (P) vs Nitrate (U)	0.680	0.044	9
Nitrate (P) vs DMA (U)	0.731	0.025	9
Nitrate (P) vs Nitrate (U)	0.810	0.008	9
$P_{NOx}R$ vs Nitrate (U)	0.775	0.014	9
ADMA (U) vs DMA (U)	0.685	0.042	9
ADMA (U) vs Nitrate (U)	0.927	< 0.001	9
ADMA (U) vs Nitrite (U)	0.716	0.030	9
DMA (U) vs Nitrate (U)	0.809	0.008	9
DMA (U) vs Nitrite (U)	0.675	0.046	9
Nitrate (U) vs Nitrite (U)	0.735	0.024	9

Abbreviations. Arg, Arginine; ADMA, asymmetric dimethylamine; Arg/ADMA, arginine/asymmetric dimethylarginine ratio; DMA, dimethylamine; DMA/ADMA, dimethylamine/asymmetric dimethylarginine ratio; (P), plasma; $P_{NOx}R$, nitrate/nitrite ratio in plasma; (S), sputum; $S_{NOx}R$, nitrate/nitrite ratio in sputum; (U), urine; $U_{NOx}R$, nitrate/nitrite ratio in urine.

Table S3. Summary of the results of correlation analyses between members of the Arg/NO pathway in plasma (P) and urine (U) in the controls of the study.

Correlation pair	Correlation coefficient	*p* value	Data pairs (*n*)
**Arg (P) vs. ADMA (P)**	**0.280**	**0.016[a]**	**73**
**Arg (P) vs. Arg/ADMA (P)**	**0.673**	**< 0.001[b]**	**73**
Arg (P) vs. Nitrat (P)	0.116	0.442[b]	46
Arg (P) vs. Nitrite (P)	-0.252	0.091[b]	46
**Arg (P) vs. $P_{NOx}R$ (P)**	**0.313**	**0.034[b]**	**46**
Arg (P) vs. ADMA (U)	0.237	0.074[a]	59
Arg (P) vs. DMA (U)	-0.020	0.888[b]	51
Arg (P) vs. DMA/ADMA (U)	-0.173	0.225[b]	51
Arg (P) vs. Nitrat (U)	-0.128	0.334[b]	59
Arg (P) vs. Nitrite (U)	0.157	0.236[b]	59
Arg (P) vs. $U_{NOx}R$	-0.150	0.258[b]	59
**ADMA (P) vs. Arg/ADMA (P)**	**-0.403**	**< 0.001[b]**	**73**
ADMA (P) vs. Nitrat (P)	0.159	0.292[b]	46
ADMA (P) vs. Nitrite (P)	0.184	0.221[b]	46
ADMA (P) vs. $P_{NOx}R$ (P)	-0.057	0.708[b]	46
**ADMA (P) vs. ADMA (U)**	**0.464**	**< 0.001[a]**	**59**
ADMA (P) vs. DMA (U)	-0.049	0.586[b]	50
**ADMA (P) vs. DMA/ADMA (U)**	**-0.325**	**0.021[b]**	**50**
ADMA (P) vs. Nitrat (U)	-0.007	0.957[b]	59
**ADMA (P) vs. Nitrite (U)**	**0.373**	**0.004[b]**	**58**
**ADMA (P) vs. $U_{NOx}R$**	**-0.353**	**0.007[b]**	**58**
Nitrat (P) vs. Arg/ADMA (P)	-0.001	0.993[b]	46
Nitrat (P) vs. Nitrite (P)	0.038	0.802[b]	46
**Nitrat (P) vs. $P_{NOx}R$ (P)**	**0.633**	**< 0.001[b]**	**46**
Nitrat (P) vs. ADMA (U)	0.113	0.519[b]	35
Nitrat (P) vs. DMA (U)	0.042	0.820[b]	32
Nitrat (P) vs. DMA/ADMA (U)	0.025	0.892[b]	32
Nitrat (P) vs. Nitrat (U)	0.250	0.147[b]	35
Nitrat (P) vs. Nitrite (U)	-0.014	0.934[b]	35
Nitrat (P) vs. $U_{NOx}R$	0.246	0.154[b]	35
**Nitrite (P) vs. Arg/ADMA (P)**	**-0.402**	**0.006[b]**	**46**
**Nitrite (P) vs. $P_{NOx}R$ (P)**	**-0.708**	**< 0.001[b]**	**46**
Nitrite (P) vs. ADMA (U)	0.099	0.570[b]	35
Nitrite (P) vs. DMA (U)	-0.221	0.224[b]	32
Nitrite (P) vs. DMA/ADMA (U)	-0.271	0.134[b]	32
Nitrite (P) vs. Nitrat (U)	-0.080	0.647[b]	35
**Nitrite (P) vs. Nitrite (U)**	**0.353**	**0.038[b]**	**35**
**Nitrite (P) vs. $U_{NOx}R$**	**-0.380**	**0.025[b]**	**35**
**Arg/ADMA (P) vs. $P_{NOx}R$ (P)**	**0.382**	**0.009[b]**	**46**
Arg/ADMA (P) vs. ADMA (U)	-0.064	0.632[b]	58
Arg/ADMA (P) vs. DMA (U)	-0.001	0.992[b]	50
Arg/ADMA (P) vs. DMA/ADMA (U)	0.129	0.374[b]	50
Arg/ADMA (P) vs. Nitrat (U)	0.002	0.988[b]	58

Arg/ADMA (P) vs. Nitrite (U)	-0.054	0.687[b]	58
Arg/ADMA (P) vs. $U_{NOx}R$	0.096	0.474[b]	58
$P_{NOx}R$ (P) vs. ADMA (U)	0.032	0.854[b]	35
$P_{NOx}R$ (P) vs. DMA (U)	0.228	0.209[b]	32
$P_{NOx}R$ (P) vs. DMA/ADMA (U)	0.232	0.201[b]	32
$P_{NOx}R$ (P) vs. Nitrat (U)	0.249	0.150[b]	35
$P_{NOx}R$ (P) vs. Nitrite (U)	-0.263	0.127[b]	35
**$P_{NOx}R$ (P) vs. $U_{NOx}R$**	**0.449**	**0.007[b]**	**35**
ADMA (U) vs. DMA (U)	0.245	0.076[b]	53
ADMA (U) vs. DMA/ADMA (U)	-0.255	0.065[b]	53
ADMA (U) vs. Nitrat (U)	0.249	0.051[b]	62
**ADMA (U) vs. Nitrite (U)**	**0.367**	**0.004[b]**	**61**
ADMA (U) vs. $U_{NOx}R$	-0.199	0.125[b]	61
**DMA (U) vs. DMA/ADMA (U)**	**0.811**	**< 0.001[b]**	**53**
DMA (U) vs. Nitrat (U)	0.155	0.268[b]	53
DMA (U) vs. Nitrite (U)	-0.261	0.059[b]	53
DMA (U) vs. $U_{NOx}R$	0.274	0.047[b]	53
DMA/ADMA (U) vs. Nitrat (U)	-0.012	0.929[b]	53
**DMA/ADMA (U) vs. Nitrite (U)**	**-0.482**	**< 0.001[b]**	**53**
**DMA/ADMA (U) vs. $U_{NOx}R$**	**0.381**	**0.005[b]**	**53**
**Nitrat (U) vs. Nitrite (U)**	**0.359**	**0.004[b]**	**61**
Nitrat (U) vs. $U_{NOx}R$	0.215	0.096[b]	61
**Nitrite (U) vs. $U_{NOx}R$**	**-0.785**	**< 0.001[b]**	**61**

Abbreviations. Arg, arginine; ADMA, asymmetric dimethylarginine; DMA, dimethylamine; P, plasma; U, urine; $P_{NOx}R$, plasma nitrate/nitrite ratio; $U_{NOx}R$, urinary nitrate/nitrite ratio; [a]Pearson correlation; [b]Spearman correlation. Significant results are marked in bold.
